# Supplementary material for: APOL1 genotype associated risk for preeclampsia in African populations: Rationale and protocol design for studies in women of African ancestry in resource limited settings
Source: PLoS One. 2022 Dec 29;17(12):e0278115. doi: 10.1371/journal.pone.0278115 (PMC9799323; doi:10.1371/journal.pone.0278115)
Supplement: Attachment — Submitted filename: response to reviewers.pdf [file pone.0278115.s002.pdf]

October 18, 2022

Dear Dr. Antonello,

Thank you for all the reviews,  the input has been valuable in improving the manuscript.
Please find below our responses to the reviewers comments. Our responses are marked red in the document.

Yours sincerely,

C. Osafo

Charlotte Osafo MBCHB, FWACP, FGCPS, MS

Review Comments to the Author

Reviewer #1: The authors have addressed this reviewer's comments. I have only two minor edits: APOL1 when referring to the gene is always italicized; when referring to the protein it is not. Please correct in abstract and throughout text

We have italicised all APOL1 genes

Preeclampsia is a disease and is therefore not capitalized in abstract and elsewhere.

We have done as suggested.

Reviewer #2: I believe the authors have addressed the reviewer's initial comments satisfactorily. There are two areas that could use some additional clarification (but not re-review). First, limiting the sample to women without a history of preeclampsia removes from the population women who might have recurrent preeclampsia due to their (or their fetus's) APOL1 status. This would result in an underestimate of the relationship between apol1 and preeclampsia generally, and is worthy of a statement to this regard in the discussion.

We have included a statement to that effect "This may result in an underestimate of the relationship between APOL1 and preeclampsia".

Second, there is still a little confusion in the manuscript about when the maternal blood draw occurs. According to table 2, biomarkers and blood samples are taken at recruitment, but I believe that they are actually taken at delivery (as stated in the section above entitled 'Sample collection and laboratory assays'. it would be good to go thru the manuscript one last time and clarify exactly when these samples are obtained.

We have taken a look and we are ok with the version displayed in the mansucript
